# High-Throughput B Cell Epitope Determination by Next-Generation Sequencing

**DOI:** 10.3389/fimmu.2022.855772

**Published:** 2022-03-23

**Authors:** Lauren M. Walker, Andrea R. Shiakolas, Rohit Venkat, Zhaojing Ariel Liu, Steven Wall, Nagarajan Raju, Kelsey A. Pilewski, Ian Setliff, Amyn A. Murji, Rebecca Gillespie, Nigel A. Makoah, Masaru Kanekiyo, Mark Connors, Lynn Morris, Ivelin S. Georgiev

**Affiliations:** ^1^ Vanderbilt Vaccine Center, Vanderbilt University Medical Center, Nashville, TN, United States; ^2^ Department of Pathology, Microbiology and Immunology, Vanderbilt University Medical Center, Nashville, TN, United States; ^3^ Vaccine Research Center, National Institute of Allergy and Infectious Diseases, National Institutes of Health (NIH), Bethesda, MD, United States; ^4^ Division of Virology, Faculty of Health Sciences, University of the Free State, Bloemfontein, South Africa; ^5^ National Institute for Communicable Diseases of the National Health Laboratory Service, Johannesburg, South Africa; ^6^ National Institute of Allergy and Infectious Diseases, National Institutes of Health (NIH), Bethesda, MD, United States; ^7^ Antibody Immunity Research Unit, Faculty of Health Sciences, University of the Witwatersrand, Johannesburg, South Africa; ^8^ Centre for the AIDS Programme of Research in South Africa (CAPRISA), University of KwaZulu-Natal, Durban, South Africa; ^9^ Vanderbilt Institute for Infection, Immunology, and Inflammation, Vanderbilt University Medical Center, Nashville, TN, United States; ^10^ Department of Electrical Engineering and Computer Science, Vanderbilt University, Nashville, TN, United States; ^11^ Center for Structural Biology, Vanderbilt University, Nashville, TN, United States; ^12^ Program in Computational Microbiology and Immunology, Vanderbilt University Medical Center, Nashville, TN, United States

**Keywords:** single cell, epitope, monoclonal antibody, HIV, next generation sequencing (NGS)

## Abstract

Development of novel technologies for the discovery of human monoclonal antibodies has proven invaluable in the fight against infectious diseases. Among the diverse antibody repertoires elicited by infection or vaccination, often only rare antibodies targeting specific epitopes of interest are of potential therapeutic value. Current antibody discovery efforts are capable of identifying B cells specific for a given antigen; however, epitope specificity information is usually only obtained after subsequent monoclonal antibody production and characterization. Here we describe LIBRA-seq with epitope mapping, a next-generation sequencing technology that enables residue-level epitope determination for thousands of single B cells simultaneously. By utilizing an antigen panel of point mutants within the HIV-1 Env glycoprotein, we identified and confirmed antibodies targeting multiple sites of vulnerability on Env, including the CD4-binding site and the V3-glycan site. LIBRA-seq with epitope mapping is an efficient tool for high-throughput identification of antibodies against epitopes of interest on a given antigen target.

## Introduction

Due to their high target specificity and relatively low incidence of adverse effects, monoclonal antibody therapeutics have become a mainstay in treatment options for a variety of human diseases such as infection, cancer, autoimmunity, and a multitude of others (1–3). For the last several decades antibodies have been used effectively in the clinic to treat patients and one area where considerable effort has gone into developing antibodies for treatment and prevention of infection is HIV-1 (4–12). Although there has been considerable progress made in drugs used to treat infection such as anti-retrovirals (ART), the lack of a licensed HIV-1 vaccine and the emergence of resistance to current ART regimes emphasize the need for novel treatment options (13–17). Antibodies represent an attractive therapeutic option in this context, and as such, multiple antibodies are currently being investigated in clinical trials (18–30). All antibodies being tested in the clinic target the HIV-1 envelope glycoprotein (Env), the sole target for neutralizing antibodies (31, 32). There are multiple antigenic regions on Env that have been shown to be important epitopes targeted by neutralizing antibody responses, including the CD4-binding site (CD4bs), glycan-dependent epitopes (V1V2 and V3), membrane-proximal external region (MPER), gp120-gp41 interface, and others (33–44).

Technologies to discover and develop new antibody therapeutics such as hybridoma generation, antigen-specific B cell sorting, phage or yeast display, and single-cell B cell sequencing, have made the process generally quicker and more efficient (45–50). An underlying goal of antibody discovery technologies is to identify paired heavy and light chain B cell receptor (BCR) sequences from antigen-specific B cells. This is then followed up by subsequent antibody production and characterization. However, only limited information about antigen and epitope specificity is obtained during initial B cell or antibody screening steps, often requiring the profiling of tens to hundreds of antibodies to identify the few with epitopes or functions of interest. In order to overcome these obstacles, we recently developed LIBRA-seq, a next-generation single-cell sequencing technology for simultaneous recovery of BCR sequence and antigen specificity by utilizing DNA-barcoded antigens (51). LIBRA-seq has successfully been applied to identify antibodies against infectious agents such as HIV-1, influenza, and a number of coronaviruses (51–53).

Here, we aimed to increase the power and utility of LIBRA-seq to include simultaneous recovery of B cell receptor sequence and paired antigen epitope specificity at the level of individual epitope residues. To this end, we developed a panel of DNA-barcoded HIV-1 Env epitope-specific variants, to distinguish epitope-specific B cells, and allowing for residue-level epitope binding information to be transformed into a sequence-able event. We applied LIBRA-seq with epitope mapping to samples from chronically-infected HIV-1 donors, leading to the discovery of antibodies specific to multiple different Env epitope regions, including the CD4bs and V3-glycan sites. In addition, we utilized an antigen panel of pre-fusion stabilized Env gp140 trimers along with monomeric gp120 proteins to identify conformation-dependent B cells, allowing domain-level epitope binding information to be transformed into a sequence-able event. LIBRA-seq with epitope mapping is a high-throughput antibody discovery platform that enables the identification of residue-level epitope information through next-generation sequencing, therefore reducing the burden of epitope-specific antibody discovery away from the costly and laborious subsequent steps of antibody production and characterization.

## Materials and Methods

### Human Subjects

HIV-1 infected donors were enrolled in investigational review board approved clinical protocols at the National Institutes of Allergy and Infectious Diseases (NIAID). Peripheral blood mononuclear cells (PBMCs) were collected from donors NIAID26 (N26), NIAID27 (N27), NIAID55 (N55), NIAID76 (N76) and NIAID16 (N16). Collection dates were as follows: 9/30/2009, 6/16/2006, 11/6/2008, 12/12/2007 and 7/2/2007. All donors were chronically infected at the time of sample collection. It is likely all patients were infected with clade B strain of HIV-1.

### Antigen Production

In total, four LIBRA-seq experiments were performed. In one experiment, seven HIV-1 gp140 BG505.SOSIP variants were used: single-chain BG505sc.SOSIP.T332N (backbone sequence for all BG505.SOSIP variants), BG505sc.SOSIP.N332T, BG505sc.SOSIP.N279K, BG505sc.SOSIP.D368R/N279K, BG505sc.SOSIP.N160K, BG505sc.SOSIP.K169E, BG505sc.SOSIP.D368R, and one Influenza hemagglutinin variant A/New Caledonia/20/99 H1N1 (NC99) (54–56). These antigens were used to identify antigen-positive B cells from peripheral blood mononuclear cells (PBMCs) from donors N16 and N76. In experiment 2, the antigen panel from experiment 1 was used, with the following modifications: BG505.6R.SOSIP.T332N replaced BG505sc.SOSIP.T332N, seven HIV-1 gp140 CZA97.SOSIP variants were added (CZA97sc.SOSIP.N332T, CZA97sc.SOSIP.N160K, CZA97sc.SOSIP.K169E, CZA97sc.SOSIP.N279K, CZA97sc.SOSIP.D368R/N279K, CZA97sc.SOSIP.D368R, CZA97.6R.SOSIP.T332N (backbone sequence for all CZA97.SOSIP variants) (57), and hepatitis C antigen JFH-1 E2c was also added. These antigens were used to sort antigen positive B cells from PBMCs from donor N55. In the final two LIBRA-seq experiments, HIV-1 gp140 SOSIP antigens CZA97, ZM197 and CNE55 were utilized along with HIV-1 gp120 proteins, A244 and ConC, and influenza hemagglutinin variants, A/Michigan/45/2015 H1N1, A/Indonesia/5/2005 H5N1, A/Anhui/1/2013 H7N9, H9 Hong Kong 2009 HA, and H10 Jiangxi-Donghu 2013 HA. These antigens were used to sort antigen positive B cells from donors N26 and N27.

Recombinant HIV-1 trimer and HCV proteins were expressed in Expi 293F mammalian cells (Thermo Fisher) with PEI transfection reagent and cultured for 5-7 days in FreeStyle F17 expression Medium supplemented (Thermo Fisher) with 10% Pluronic acid and 20% glutamine. Cells were maintained at 37°C with 8% CO_2_ saturation while shaking. After 5-7 days cells were spun down and supernatant was harvested. Supernatant was run over an affinity column of agarose-bound Galanthus nivalis lectin (GNA, Snowdrop). The column was washed with 1X PBS and bound protein was eluted with 1M methyl-a-D-mannopyranoside. Protein elute was then buffer exchanged into 1X PBS and further purified by size exclusion chromatography with a Superdex 200 Increase 10/300 GL Sizing column on the AKTA FPLC system (GE Life Sciences). Purified protein was analyzed by SDS-PAGE and characterized by ELISA.

Recombinant ConC gp120 and A244 gp120 with an Avi-tag at the C-terminus were expressed in HEK 293 F cells using PEI-max (Polysciences) for 72h. Culture supernatant was harvested and centrifuged to remove cells and cell debris. The cleared supernatant was filtered through 0.22 stericup filters (Millipore) and the soluble gp120 proteins were then purified by affinity chromatography by passing the supernatant through a column of agarose bound *Glanathus nivalus lectin* (Sigma). The column was then washed with 1x PBS and proteins were eluted with 1 M methyl α-D-mannopyranoside (Sigma). The eluted proteins were concentrated using a 30 KDa cut-off vivaspin filters and further purified by ion exchange chromatography on a HiPrep™ Q HP 16/10 anion exchange column. The gp120 fraction was then collected and buffer exchanged and the proteins concentrated using a 30 Kda cut-off vivaspin filter. The purified proteins were tested for antigenicity by ELISA and the purity was checked by SDS-PAGE.

Recombinant HA proteins (A/New Caledonia/20/99 H1N1 GenBank ACF41878 (NC99), A/Michigan/45/2015 H1N1 GenBank AMA11475, A/Indonesia/5/2005 H5N1 GenBank ABP51969, A/Anhui/1/2013 H7N9 GISAID EPI439507, A/Hong Kong/33982/2009 H9N2 GISAID EPI470900, and A/Jaingxi-Donghu/246/2013 H10N8 GISAID EPI497477) all contained the HA ectodomain with a point mutation at the sialic acid-binding site (Y98F), T4 fibritin foldon trimerization domain, AviTag, and hexahistidine-tag, and were expressed in Expi 293F mammalian cells using Expifectamine 293 transfection reagent (Thermo Fisher Scientific) and cultured for 4-5 days. Culture supernatant was harvested and cleared as above, and then adjusted pH and NaCl concentration by adding 1M Tris-HCl (pH 7.5) and 5M NaCl to 50 mM and 500 mM, respectively. Ni Sepharose excel resin (GE Healthcare) was added to the supernatant to capture hexahistidine tag. Resin was separated on a column by gravity and captured HA protein was eluted by a Tris-NaCl (pH 7.5) buffer containing 300 mM imidazole. The elute was further purified by a size exclusion chromatography with a HiLoad 16/60 Superdex 200 column (GE Healthcare). Fractions containing HA were concentrated, analyzed by SDS-PAGE and tested for antigenicity by ELISA with known antibodies. Proteins were frozen at -80C until use.

AviTagged antigens were biotinylated using BirA biotin ligase (Avidity LLC).

### DNA-Barcoding of Antigens

We used oligos that possess 15 bp antigen barcode, a sequence capable of annealing to the template switch oligo that is part of the 10X bead-delivered oligos and contain truncated TruSeq small RNA read 1 sequences in the following structure: 5’-CCTTGGCACCCGAGAATTCCANNNNNNNNNNNNNCCCATATAAGA*A*A-3’, where Ns represent the antigen barcode. For each antigen, a unique DNA barcode was directly conjugated to the antigen itself. In particular, 5’amino-oligonucleotides were conjugated directly to each antigen using the Solulink Protein-Oligonucleotide Conjugation Kit (TriLink cat no. S-9011) according to manufacturer’s instructions. Briefly, the oligo and protein were desalted, and then the amino-oligo was modified with the 4FB crosslinker, and the biotinylated antigen protein was modified with S-HyNic. Then, the 4FB-oligo and the HyNic-antigen were mixed together. This causes a stable bond to form between the protein and the oligonucleotide. The concentration of the antigen-oligo conjugates was determined by a BCA assay, and the HyNic molar substitution ratio of the antigen-oligo conjugates was analyzed using the NanoDrop according to the Solulink protocol guidelines. AKTA FPLC was used to remove excess oligonucleotide from the protein-oligo conjugates, which were also verified using SDS-PAGE with a silver stain. Antigen-oligo conjugates were also used in flow cytometry titration experiments. The following barcodes were used for the donors N16 and N76 experiments: ATTCGCCTTACGCAA (BG505sc.SOSIP.T332N), AACCCACCGTTGTTA (BG505sc.SOSIP.N332T), GGTAGCCCTAGAGTA (BG505sc.SOSIP.N279K), CAGTAAGTTCGGGAC (BG505sc.SOSIP.DKO), CTTCACTCTGTCAGG (BG505sc.SOSIP.N160K), TACGCCTATAACTTG (BG505sc.SOSIP.K169E), AGACTAATAGCTGAC (BG505sc.SOSIP.D368R), and GCTCCTTTACACGTA [A/New Caledonia/20/99 H1N1 (NC99)]. The following barcodes were used for the donor N55 experiment: GCAGCGTATAAGTCA (BG505sc.SOSIP.T332N), GCTCCTTTACACGTA (BG505sc.SOSIP.N332T), CTTCACTCTGTCAGG (BG505sc.SOSIP.N279K), TGGTAACGACAGTCC (BG505sc.SOSIP.DKO), TGTGTATTCCCTTGT (BG505sc.SOSIP.N160K), TACGCCTATAACTTG (BG505sc.SOSIP.K169E), GTGTGTTGTCCTATG (BG505sc.SOSIP.D368R), AACCCACCGTTGTTA [A/New Caledonia/20/99 H1N1 (NC99)]; CAGTAAGTTCGGGAC, GTAAGACGCCTATGC, TTTCAACGCCCTTTC, CCGTCCTGATAGATG, TCATTTCCTCCGATT, GGTAGCCCTAGAGTA, AGACTAATAGCTGAC (CZA97 antigens). The following barcodes were used for the donors N26 and N27 experiments: CAGCCCACTGCAATA (CZA97), ATCGTCGAGAGCTAG (ZM197), TCACAGTTCCTTGGA (CNE55), CAGATGATCCACCAT (A244), GACCTCATTGTGAAT (ConC), TGACCTTCCTCTCCT (A/Michigan/45/2015 H1N1), CAGGTCCCTTATTTC (A/Indonesia/5/2005 H5N1), ACAATTTGTCTGCGA (A/Anhui/1/2013 H7N9), AACCTTCCGTCTAAG (H9 Hong Kong 2009 HA), AATCACGGTCCTTGT (H10 Jiangxi-Donghu 2013 HA).

### Antigen-Specific B Cell Sorting

For each sample, PBMCs were mixed with DNA-barcoded fluorescently labeled antigens, stained with fluorescent cell markers and single cell sorted using fluorescence-activated cell sorting (FACS). Briefly, cells were thawed and washed twice with DPBS 0.1% BSA. For donors NIAID16 and NIAID76 cells were stained with antibodies against cell markers including viability dye (Ghost Red 780), CD14-APC-Cy7, IgM-APC-Cy7, CD3-FITC, CD19-BV711, and IgG-PE-Cy5. For donor NIAID55 cells were stained with antibodies against cell markers including: CD3-APC-Cy7, IgG-FITC, CD19-BV711, and CD14-V500. For donors NIAID26 and NIAID27 cells were stained with antibodies against cell markers including viability dye (Ghost Red 780), CD14-APC-Cy7, CD3-FITC, CD19-BV711, and IgG-PE-Cy5. The cell-antigen mixture was incubated in the dark for 15-30 minutes. The cells were then washed twice with DPBS 0.1% BSA and stained with Streptavidin-PE (1:1000) for 15-30 minutes in the dark. The cells were washed again twice with DPBS 0.1% BSA and taken to the Flow Cytometry core for single cell sorting by FACS. Antigen positive cells were collected and delivered to the sequencing core VANTAGE for single-cell processing and sequencing.

### Sample Preparation, Library Preparation, and Sequencing

Single-cell suspensions were loaded onto the Chrommium microfluidics device (10X Genomics) and processed using the B-cell VDJ solution according to manufacturer’s suggestions. Minor modifications were made in order to separate the antigen and cellular mRNA barcode libraries as previously described (51).

### Sequence Processing and Bioinformatics Analysis

Paired-end FASTQ files of oligo libraries were used as input and processed using a previously described pipeline (52). Reads for cell barcode, UMI and antigen barcodes were used to generate a cell barcode-antigen barcode UMI count matrix. BCR contigs were processed using Cell Ranger (10X Genomics) using GRCh38 as reference. Antigen barcode libraries were also processed using Cell Ranger (10X Genomics). The overlapping cell barcodes between the two libraries were used as the basis of the subsequent analysis. We removed cell barcodes that had only non-functional heavy chain sequences as well as cells with multiple functional heavy chain sequences and/or multiple functional light chain sequences. Additionally, we aligned the BCR contigs (filtered_contigs.fasta file output by Cell Ranger, 10X Genomics) to IMGT reference genes using HighV-Quest. The output of HighV-Quest was parsed using ChangeO and merged with an antigen barcode UMI count matrix. In experiments utilizing the panel of DNA-barcoded HIV-1 Env epitope-specific variants, the LIBRA-seq score was determined as in (58). In experiments utilizing stabilized Env trimers ZM197, CNE55, CZA97, along with monomeric gp120 proteins from strains A244 and ConC, the LIBRA-seq score was determined as in (51). Next, to prioritize BCR sequences for recombinant antibody production and characterization, further filtering steps were applied; cells with UMI scores for negative control antigens > 20 and cells for which the max antigen UMI ≤ 10 were not considered for further analysis. Due to consistently poor UMI signal for the CZA97.SOSIP backbone across cells, the data for CZA97.SOSIP variants for donor N55 was not included in further analysis.

### Antibody Expression and Purification

For each antibody, variable gene sequences were inserted into custom plasmids encoding the heavy chain IgG_1_ constant region and the corresponding lamda or kappa light chain region (pTwist 314 CMV BetaGlobin WPRE Neo vector, Twist Bioscience). Antibodies were expressed in Expi293F mammalian cells (Thermo Fisher) with PEI transfection reagent and cultured for 5-7 days in FreeStyle F17 expression Medium supplemented with 10% Pluronic acid and 20% glutamine. Cells were maintained at 37°C with 8% CO2 saturation while shaking. After 5-7 days cells were spun down and supernatant was harvested. Supernatant was run over a protein A affinity column. The column was washed with 1X PBS and the protein was eluted with 100 mM Glycine HCl at 2.7 pH directly into a 1:10 volume of 1M Tris-HCl pH 8.0. Eluted antibodies were buffer exchanged using Amicon Ultra-centrifugal filter units into 1X PBS and stored for future use.

### ELISA

To assess antibody binding, soluble protein was plated on Immulon 2HB plates at 2 μg/mL overnight at 4°C. In cases where capture ELISA was used, plates were pre-incubated overnight at 4°C with 2μg/ml anti-AviTag (GenScript) and washed 3X with PBS+ 0.05% Tween-20 (PBS-T) before antigen plating for 2hr at room temperature (RT). Plates were washed three times with PBS supplemented with PBS-T and coated with 5% milk powder in PBS-T. Plates were incubated for one hour at RT. Plates were washed three times with PBS-T. Primary antibodies were diluted in 1% milk in PBS-T, starting at 10 μg/mL with a serial 1:10 dilution and then added to the plate. The plates were incubated at RT for one hour and then washed three times in PBS-T. The secondary antibody, goat anti-human IgG conjugated to peroxidase (Thermo Fisher), was added at 1:10,000 dilution in 1% milk in PBS-T to the plates, which were incubated for one hour at RT. Plates were washed three times with PBS-T and then developed by adding TMB substrate (Thermo Fisher) to each well. The plates were incubated at room temperature for ten minutes, and then 1N sulfuric acid was added to stop the reaction. Plates were read at 450 nm. Data are represented as either mean ± SEM for one ELISA experiment or %AUC normalized to BG505.SOSIP.backbone. ELISAs were repeated 2 or more times. The area under the curve (AUC) was calculated using Prism software version 8.0.0 (GraphPad).

### CD4 Binding Inhibition Assay

96-well plates were coated with 2 μg/mL purified recombinant BG505sc.backbone at 4°C overnight. The next day, plates were washed three times with PBS-T and coated with 5% milk powder in PBS-T. Plates were incubated for one hour at RT and then washed three times with PBS-T. Purified antibodies were diluted in blocking buffer at 50 μg/mL in duplicate, added to the wells, and incubated at RT. Without washing, biotinylated recombinant human CD4-Fc tag protein (Sino Biological) was added to wells for a final 20 μg/mL concentration of CD4-Fc and incubated for 30 minutes at RT. Plates were washed three times with PBS-T, and bound CD4-Fc was detected using Streptavidin-HRP (Thermo Fisher) and TMB substrate. The plates were incubated at room temperature for ten minutes, and then 1N sulfuric acid was added to stop the reaction. Plates were read at 450nm. CD4-Fc binding without antibody served as a control. Experiments were done in biological replicate and technical duplicate.

### Competition ELISA

Competition ELISA experiments were performed as above with minor modifications. After coating with antigen and blocking, non-biotinylated competitor antibody was added to each well at 20 μg/ml and incubated at RT for 30mins. After washing, biotinylated antibody (final concentration of 2 μg/ml) was added and incubated for 30mins at RT. After washing three times with PBS-T, streptavidin-HRP was added at 1:10,000 dilution in 1% milk powder in PBS-T and incubated for 1 hour at room temperature. Plates were washed and substrate and sulfuric acid were added as described above.

## Results

### Targeted Identification of CD4 Binding Site and V3 Glycan Site Directed Antibodies Using LIBRA-Seq

**Figure 1:**
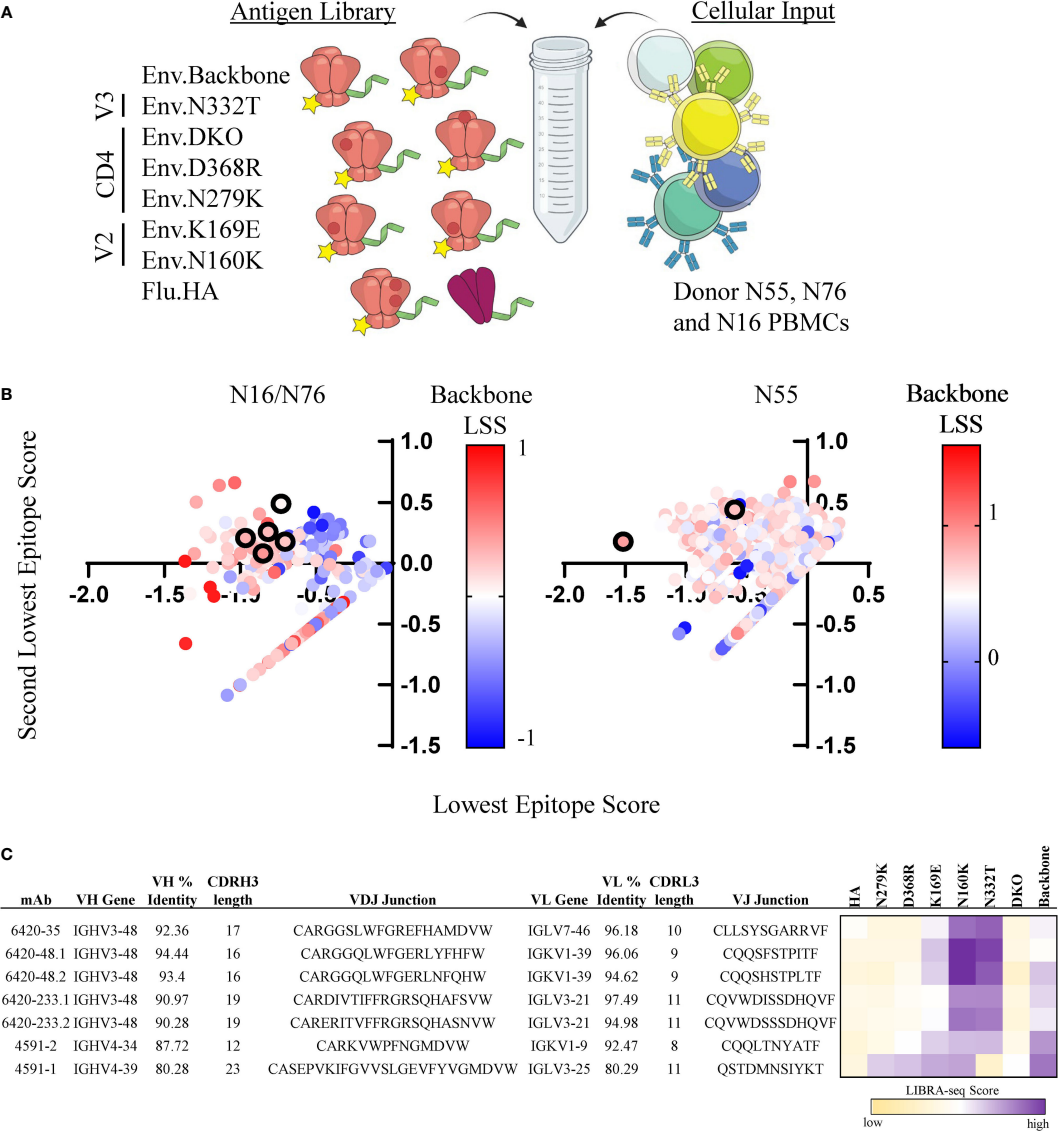
Identification of CD4bs and V3-glycan site specific antibodies from HIV-1 infected subjects. **(A)** Schematic of DNA barcoded antigens used in LIBRA-seq with epitope mapping assays. **(B)** Cells (dots) from experiments 1 (left, donors N16 and N76) and 2 (right, N55) are shown. For a given cell, the minimum LIBRA-seq score among all antigen variants for a given epitope (CD4bs, V3-glycan, V2) was computed, and the lowest (x-axis) and second-lowest (y-axis) epitope scores are plotted. Each dot is colored by the LIBRA-seq score for BG505sc.SOSIP (backbone), from lowest (blue) to highest (red). Antibodies selected for validation are shown in black outline. **(C)** Sequence characteristics and LIBRA-seq score information for candidate antibodies. Percent identity was calculated at the nucleotide level, and CDR3 length and sequences are displayed at the amino acid level. LIBRA-seq scores for each antigen are displayed on a scale of light yellow(low)-white-purple(high).

To demonstrate the feasibility of the LIBRA-seq with epitope mapping technology, we sought to identify HIV-1 Env epitope-specific antibodies from chronically infected HIV-1 donors. In two separate experiments, we screened for antigen-positive B cells using a panel of pre-fusion stabilized, soluble Env variants that contained point mutations in distinct antigenic regions on the HIV-1 Env protein (Clade A/BG505) (54–56). Each antigen was labeled with a unique DNA barcode and a fluorescent tag for fluorescence-activated cell sorting (FACS). In the screening libraries, we included a backbone Env strain (BG505.SOSIP.T332N), three CD4bs mutants (BG505.SOSIP.D368R, BG505.SOSIP.N279K, and BG505.SOSIP.D368R/N279K [referred to as double knockout (DKO)]; two V2 loop mutants (BG505.SOSIP.K169E, and BG505.SOSIP.N160K), and one V3-glycan site mutant (BG505.SOSIP.N332T) (**Figure 1A**). Together, these two experiments resulted in the identification of 2,442 B cells with antigen specificity and BCR sequence information (**Supplementary Figure 1**).

Next, we prioritized the recombinant expression of a set of B cell receptors based on the criteria that the cells displayed a distinct epitope signature across the different Env variants, such as a low LIBRA-seq score for a given epitope and high LIBRA-seq scores for the respective backbone and other epitope variants. Among the candidate B cells, we identified and selected for validation several cells with CD4bs epitope specificity signal by LIBRA-seq (**Figures 1B, C**). Specifically, we selected two lineages with two clonally related antibody members in each lineage, and two additional antibodies that did not belong to either clonal lineage. These antibodies, 6420-35, 6420-48.1, 6420-48.2, 6420-233.1, 6420-233.2 and 4591-2, displayed low LIBRA-seq scores for the BG505.SOSIP CD4bs variants and high LIBRA-seq scores for the backbone, V2 and V3-glycan BG505.SOSIP variants (**Figures 1B, C**). Five of the antibodies utilized the IGHV3-48 heavy chain variable gene and one antibody utilized the IGHV4-34 heavy chain variable gene. The antibodies displayed moderate to high levels of heavy chain somatic hypermutation (6-12%) and CDRH3 lengths of 12-19 amino acids (**Figure 1C**). The antibodies utilized several different light chain germline genes (IGLV7-46, IGKV1-39, IGLV3-21 and IGKV1-9). In addition to the predicted CD4bs antibodies, we selected for validation an additional antibody with a predicted V3-glycan epitope specificity. This antibody, 4591-1, displayed a low LIBRA-seq score for the BG505.SOSIP V3-glycan variant N332T and high LIBRA-seq scores for the BG505.SOSIP.backbone, V2 and CD4bs variants (**Figure 1B, C**). This antibody utilized variable heavy chain gene IGHV4-39 and displayed a high level of somatic hypermutation (20%) and a CDRH3 length of 22 amino acids (**Figure 1C**).

**Figure 2:**
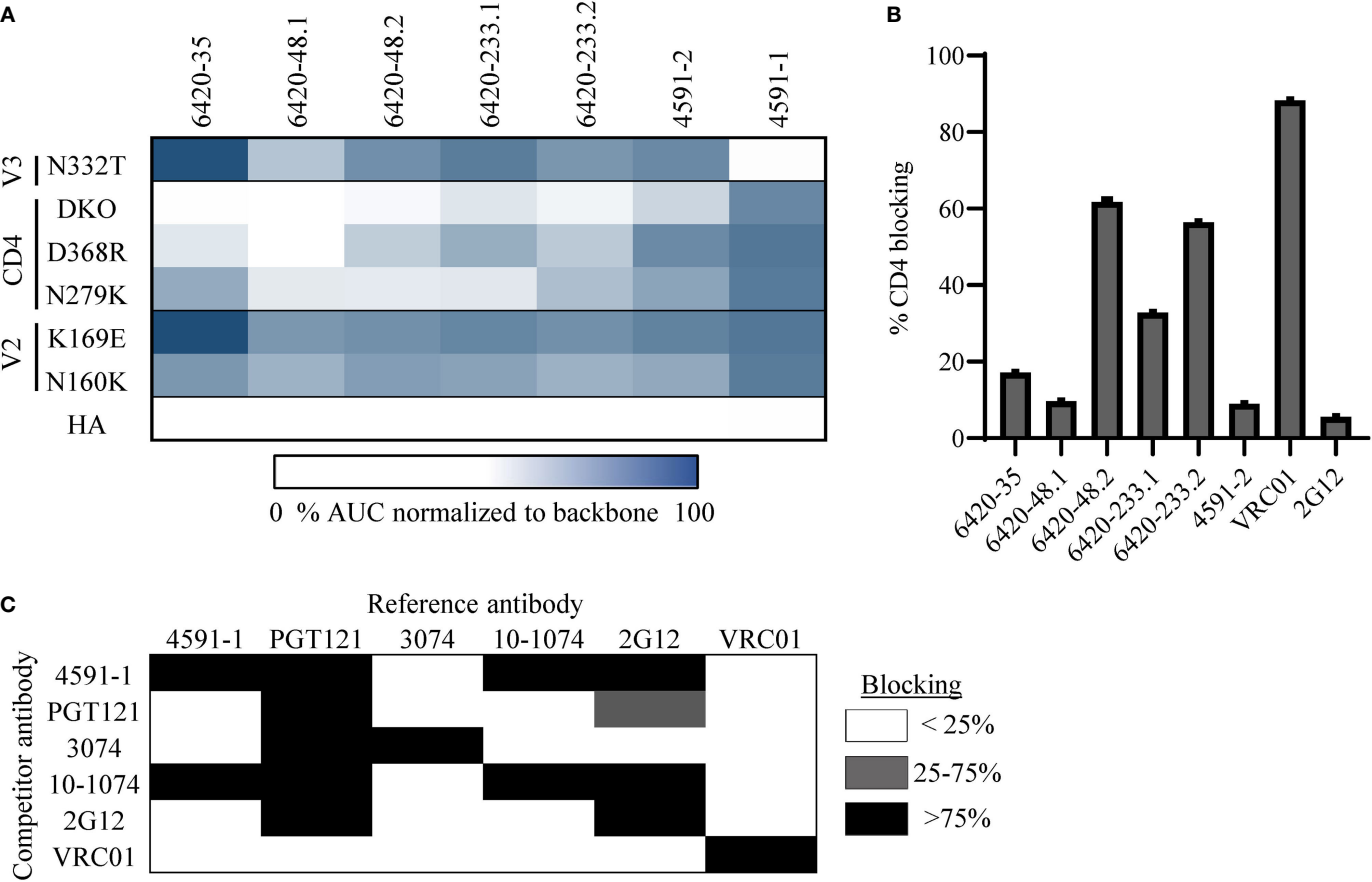
Validation and characterization of CD4bs and V3-glycan specific antibodies discovered through LIBRA-seq. **(A)** A heat map of ELISA AUC values for binding to BG505.SOSIP variant proteins are shown for each antibody. AUC values for each variant were normalized to BG505.SOSIP.backbone for each antibody. ELISA binding curves are located in the supplement. **(B)** HIV-1 Env/CD4 inhibition ELISA data is shown for all antibodies. CD4bs antibody VRC01 was used as a positive control and outer domain glycan specific antibody 2G12 was used as a negative control. Data is shown as % CD4 blocking by each antibody. **(C)** Competition ELISA of 4591-1 with antibodies PGT121, 3074, 2G12, and 10-1074. Antibody VRC01 was used as a negative control. Data is shown as % inhibition of binding by the reference antibody. White indicates <25% inhibition, grey indicates 25-75% inhibition and black represents >75% inhibition. All ELISAs were performed in technical duplicate with 2 biological duplicates; data represented as mean ± SEM.

To confirm the epitope specificity predicted by LIBRA-seq for the selected antibodies, we tested them for binding by ELISA to BG505.SOSIP. Notably, all six antibodies exhibited binding to this antigen, confirming the HIV-1 Env specificity of all of these antibodies (**Supplementary Figure 2A**). Next, we tested these antibodies against the different epitope-specific BG505.SOSIP Env variants (**Figure 2A**). The binding of antibodies 6420-35, 6420-48.1, 6420-48.2, 6420-233.1, 6420-233.2 and 4591-2 to BG505.SOSIP Env was associated with a >2-fold decrease in binding for one or more of the CD4bs variants, but not for the V2 or V3-glycan variants (**Figure 2A** and **Supplementary Figure 2A**). Further, the antibodies were tested for their ability to bind YU2 gp120 core protein, which lacks variable regions V1, V2 and V3, and its respective CD4bs mutant D368R (59). Binding to YU2 gp120 D368R was affected to a different extent for the six antibodies, suggesting that sensitivity to this specific CD4bs mutation may be context-specific (**Supplementary Figure 2B**). Finally, we tested the ability of these six antibodies to block the interaction between HIV-1 Env and its cognate receptor CD4, using a soluble, Fc-tagged CD4. The antibodies showed different levels of CD4-blocking ability by ELISA (**Figure 2B**). Together, these results suggest that these six antibodies target the CD4bs epitope on HIV-1 Env, as predicted by LIBRA-seq.

Next, we investigated the epitope specificity of antibody 4591-1, and found that antibody binding to Env was inhibited by the BG505.SOSIP V3-glycan mutation N332T, but not by mutations in the V2 or CD4bs regions, as predicted by LIBRA-seq (**Figure 2A** and **Supplementary Figure 2A**). To further confirm the 4591-1 epitope, we tested the ability of this antibody to compete for binding to BG505.SOSIP.backbone Env with known V3-glycan antibodies PGT121, 3074, 2G12 and 10-1074 (60–63). We found 4591-1 competed with antibodies PGT121, 2G12 and 10-1074, but did not compete with V3-crown specific antibody 3074 or CD4bs antibody VRC01 (**Figure 2C**). Together, these results suggest that antibody 4591-1 targets the V3-glycan epitope on HIV-1 Env, as predicted by LIBRA-seq.

Overall, these data indicate that the LIBRA-seq technology can be successfully applied to identify antibodies against specific epitopes of interest on a target antigen.

### Targeted Identification of Monomer- and Trimer-Specific Antibodies Using LIBRA-Seq

**Figure 3:**
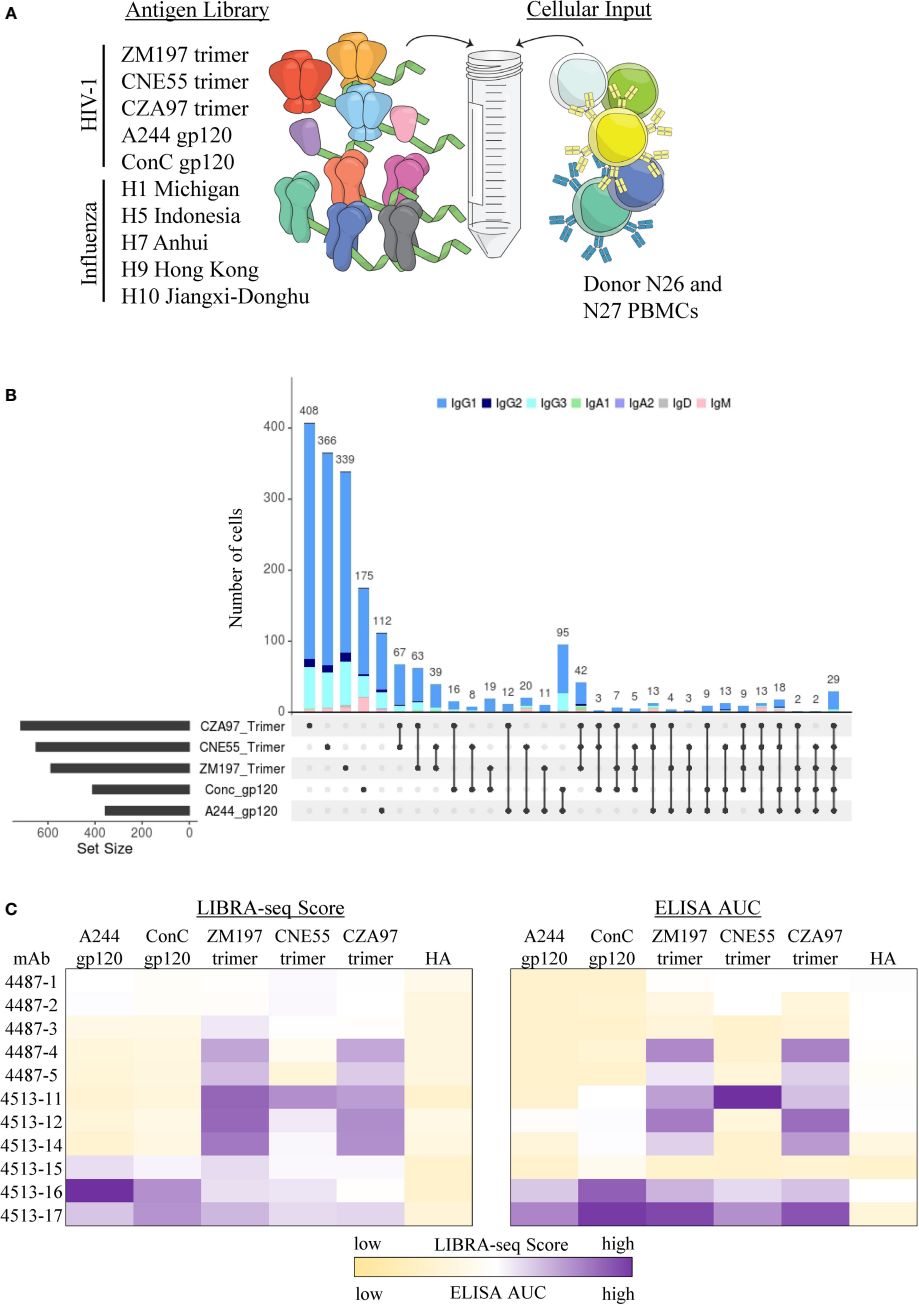
Identification of confirmational specific antibodies from LIBRA-seq with epitope mapping. **(A)** Schematic of DNA barcoded antigens used in LIBRA-seq with epitope mapping assays. **(B)** Upset plot for all B cells with LIBRA-seq score ≥1 against different HIV-1 antigen combinations. On the X-axis, each column represents a different combination of antigens (rows), showing which antigens are part of a given combination (displayed by filled circles), with the bar height corresponding to the number of B cells identified to be reactive with the given antigen combination. Each combination is mutually exclusive. The total number of B cells predictive to be reactive with each individual antigen is indicated as a horizontal bar at the bottom left of each subpanel. Isotype for each antibody is depicted by different colors. **(C)** LIBRA-seq scores and ELISA AUC values for each antibody binding to HIV-1 trimer and monomer antigens. Scores are represented by heatmaps, with the lowest scores displayed as light yellow and the highest as purple. ELISA data are representative of at least two different experiments. LIBRA-seq score data shown for H9 Hong Kong 2009 HA and ELISA AUC data shown for A/New Caledonia/20/99 H1N1 (NC99) HA. ELISA binding curves are located in the supplement.

In addition to the ability of LIBRA-seq to screen antibody candidates for residue-level epitope specificity, we sought to apply this technology toward identifying antibody candidates that specifically recognize different conformational states of the target antigen. HIV-1 Env represents an appropriate target for such an experiment since antigen variants in different conformational states are available (64). In order to determine the ability of LIBRA-seq to identify trimeric vs monomeric anti-Env antibodies, we utilized an antigen panel of pre-fusion stabilized Env gp140 trimers from multiple clade C strains (ZM197, CNE55, CZA97), along with monomeric gp120 proteins from clades AE and C (AE/A244 and C/ConC). (**Figure 3A**). These two experiments together resulted in the identification of 3,843 cells with available antigen specificity and BCR sequence information (**Supplementary Figure 3**). In particular, we observed patterns of trimer-only reactivity, monomer-only reactivity, or both monomer and trimer reactivity for a variety of B cells (**Figure 3B** and **Supplementary Figure 3**). We then prioritized two sets of B cells for recombinant monoclonal antibody production and characterization. Set one (antibodies 4513-15, 4513-16 and 4513-17) displayed positive LIBRA-seq scores for at least one trimer and at least one monomer (**Figure 3C**). Set two (antibodies 4513-11, 4513-14, 4513-12, 4487-1, 4487-2, 4487-3, 4487-4, and 4487-5) displayed positive LIBRA-seq scores for at least one trimer and low LIBRA-seq scores for the two monomers (**Figure 3C**). Interestingly, four out of the eleven antibodies, 4513-17, 4513-14, 4513-11 and 4487-5 utilized the variable heavy chain gene IGHV1-69, which has been shown to be commonly utilized during viral infections including HIV-1 (65–70), although all appeared to be from different B cell lineages (**Supplementary Figure 4A**). Overall, the eleven antibodies exhibited diverse sequence features, including the utilization of multiple heavy and light chain germline genes, a range of CDRH3 lengths, and diverse somatic mutation levels for both the heavy and light chains (**Supplementary Figure 4A**).

To confirm the conformational specificity predicted by LIBRA-seq for the selected antibodies, we tested them for binding by ELISA to the same panel of HIV-1 Env gp140 trimer and gp120 monomer antigens as used in the LIBRA-seq library (**Figure 3C**). Binding to at least one of the antigens was observed for seven of the 11 antibodies, confirming the HIV-1 Env specificity for these antibodies, as predicted by LIBRA-seq (**Figure 3C**). As suggested by the LIBRA-seq scores, antibodies 4513-16 and 4513-17 displayed binding to both monomer and trimer antigens (**Figure 3C** and **Supplementary Figure 4B**). In contrast and also as suggested by the LIBRA-seq scores, antibodies 4513-11, 4513-12, 4513-14, 4487-4 and 4487-5 displayed binding to trimeric Env protein >3 times that to monomeric Env protein, suggesting these antibodies preferentially recognize Env in its trimeric conformation state (**Figure 3C** and **Supplementary Figure 4B**). Together, these results confirm that LIBRA-seq with epitope mapping can be applied to successfully identify conformation-specific antibodies.

## Discussion

Here, we describe the application of LIBRA-seq with epitope mapping to identify novel monoclonal antibodies that target diverse sites of vulnerability on the HIV-1 Env glycoprotein. Through high-throughput sequencing experiments, we discovered and validated the specificity for several antibodies targeting the CD4bs of Env, an antibody targeting the V3-glycan site of Env, and conformation-dependent antibodies that recognize trimeric Env protein over monomeric forms of the protein. While standard epitope mapping technologies (46, 71–79) can shed light onto the epitopes targeted by antigen-specific antibodies, this is typically done either through bulk serum analysis, or for a small set of individual antibodies. In contrast, by including antigen variants with epitope-knockout mutations in the screening library, LIBRA-seq enables high-throughput, high-dimensional, single-cell epitope analysis. Further, with the generation of the paired heavy-light chain sequence information for each given antibody, LIBRA-seq analysis can also be applied to explore potential associations between antibody sequence and epitope specificity.

Despite the promising results presented here in this study, we observed certain limitations, representing opportunities for further technology improvements. In particular, consistent epitope signatures across all antigens in the panel could not be identified for a subset of the isolated antigen-specific cells. For a subset of the tested antibodies, the LIBRA-seq-predicted antigen specificity could not be confirmed, suggesting that additional optimization, such as incorporating redundancy in the antigen barcoding process, could be important to further improve the sensitivity of the technology. In addition, LIBRA-seq scores for some antigens were generally low across all cells – for example, the CZA97.SOSIP backbone used in the donor N55 sort, and as a result, epitope information for this antigen was not taken into account for subsequent analysis. These limitations could be attributed to a variety of technical factors, such as potential variations in the antigen labeling process. We also note that LIBRA-seq with epitope mapping is geared toward well characterized antigen targets where immunogenic regions of interest have been determined, and may be more challenging to adapt for studies of less well characterized pathogenic organisms or antigen targets that are associated with promiscuous B cell cross-reactivity or non-specific binding. Despite these limitations, LIBRA-seq with epitope mapping nevertheless provides an efficient strategy for the identification of epitope-specific antibodies.

Advances in antibody sequencing technologies have contributed to the increase in therapeutic antibodies available to treat patients. LIBRA-seq with epitope mapping provides critical advantages in the antibody discovery pipeline through high-resolution data generation earlier in the process. The results presented here underscore the potential of including epitope specificity as part of the sequencing readout. Prioritizing antibody candidates based on predicted epitope specificity information obtained during the initial sequencing stage can significantly decrease the burden posed by the subsequent production and validation of hundreds to thousands of potential leads for epitope-specific antibody discovery. More broadly, LIBRA-seq with epitope mapping can be applied to other disease settings where the discovery of epitope specific antibodies is the main priority or to the evaluation of vaccine candidates where understanding what types of antibodies are elicited is critical.

## Data Availability Statement

The datasets presented in this study can be found in online repositories. The names of the repository/repositories and accession number(s) can be found below: SRA, PRJNA803769 GenBank, 2547750.

## Author Contributions

Methodology, LW, AS, RV, IS, and IG. Investigation, LW, AS, RV, ZL, SW, KP, NR, IS, and AM. Software, RV, IS, and NR. Writing Original, LW. Writing review and edits, all authors. Funding acquisition, IG. Resources, RG, NM, MK, MC, LM, and IG. All authors contributed to the article and approved the submitted version.

## Funding

This work was supported in part by NIH R01 AI152693 and AI131722 (to IG).

## Conflict of Interest

IG and AS are co-founders of AbSeek Bio. The Georgiev laboratory at Vanderbilt University Medical Center has received unrelated funding from Takeda Pharmaceuticals.

LMW, ARS, RV, KAP, IS, and ISG are inventors on patent applications related to the LIBRA-seq technology and/or antibody sequences presented in this manuscript.

The remaining authors declare that the research was conducted in the absence of any commercial or financial relationships that could be construed as a potential conflict of interest.

## Publisher’s Note

All claims expressed in this article are solely those of the authors and do not necessarily represent those of their affiliated organizations, or those of the publisher, the editors and the reviewers. Any product that may be evaluated in this article, or claim that may be made by its manufacturer, is not guaranteed or endorsed by the publisher.
