# Supplementary material for: High-Throughput B Cell Epitope Determination by Next-Generation Sequencing
Source: Front Immunol. 2022 Mar 23;13:855772. doi: 10.3389/fimmu.2022.855772 (PMC8984479; doi:10.3389/fimmu.2022.855772)
Supplement: Supplementary file 1 [file Presentation_1.pdf]

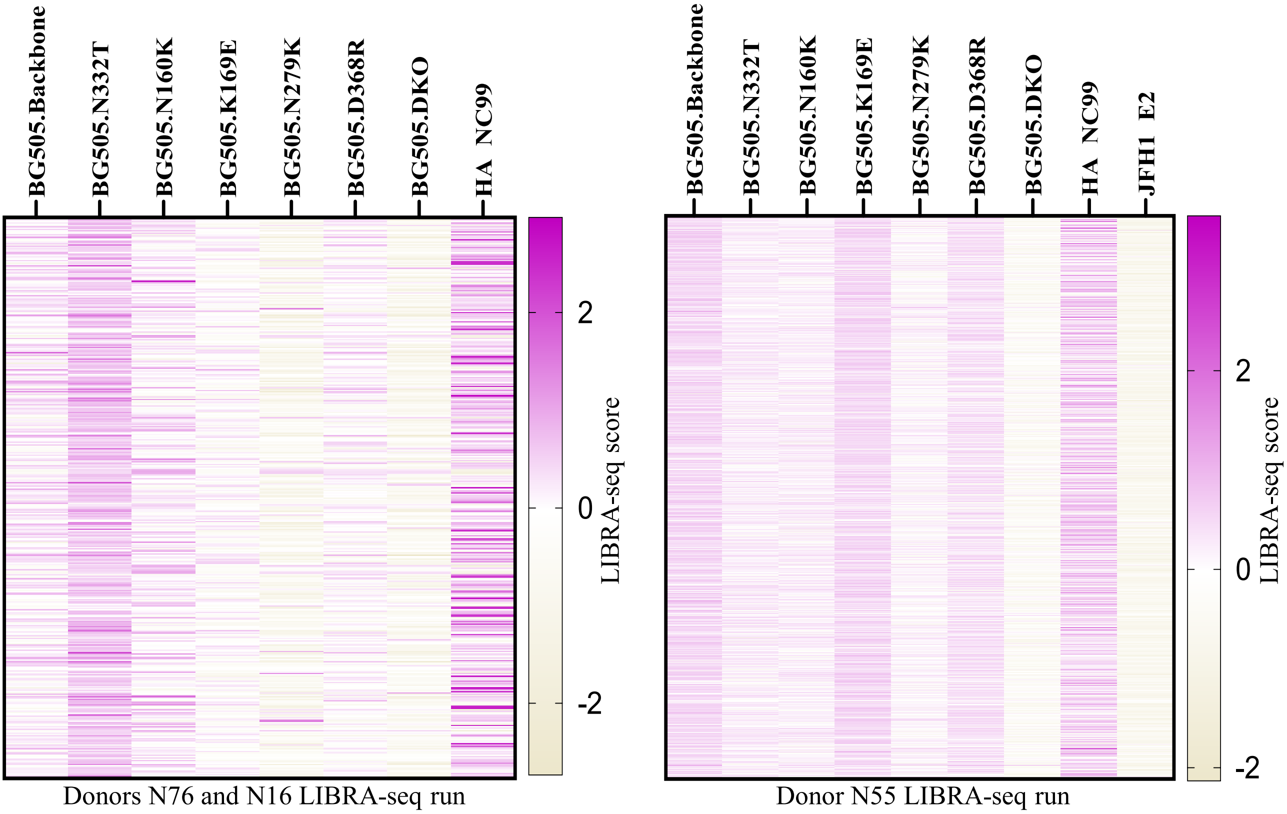

Supplementary Figure 1. Identification of HIV-1 SOSIP Specific B Cells using LIBRA-seq. LIBRA-seq score information for all cells from N76/N16 LIBRA-seq run and N55 LIBRA-seq run. LIBRA-seq score information for each antigen is displayed on a scale from light yellow(low)-white-purple(high).

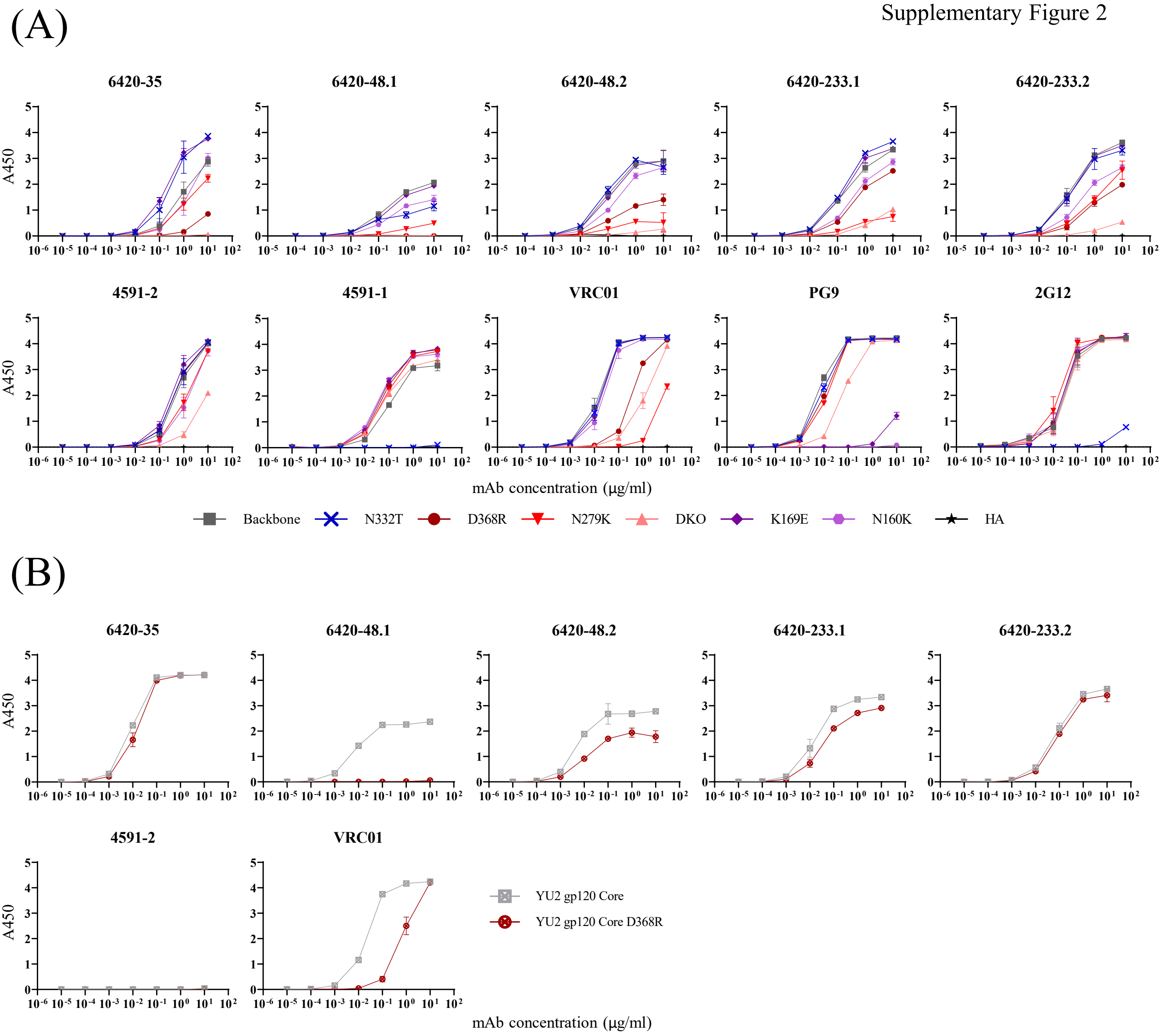

Supplementary Figure 2: Validation and characterization of CD4bs and V3-glycan specific antibodies discovered through LIBRA-seq. (A) ELISA binding curves for binding to BG505.SOSIP variant proteins are shown for antibodies: 6420-35, 6420-48.1, 6420-48.2, 6420-233.1, 6420-233.2, 4591-2 and 4591-1. Flu HA protein was used as a negative control antigen. **(B)** ELISA binding curves for binding to YU2 gp120 core and YU2 gp120 core D368R proteins are shown for antibodies: 6420-35, 6420-48.1, 6420-48.2, 6420-233.1, 6420-233.2, and 4591-2. Absorbance values at 450nm shown on Y-axis. Antibody concentration in µg/ml shown on the X-axis.

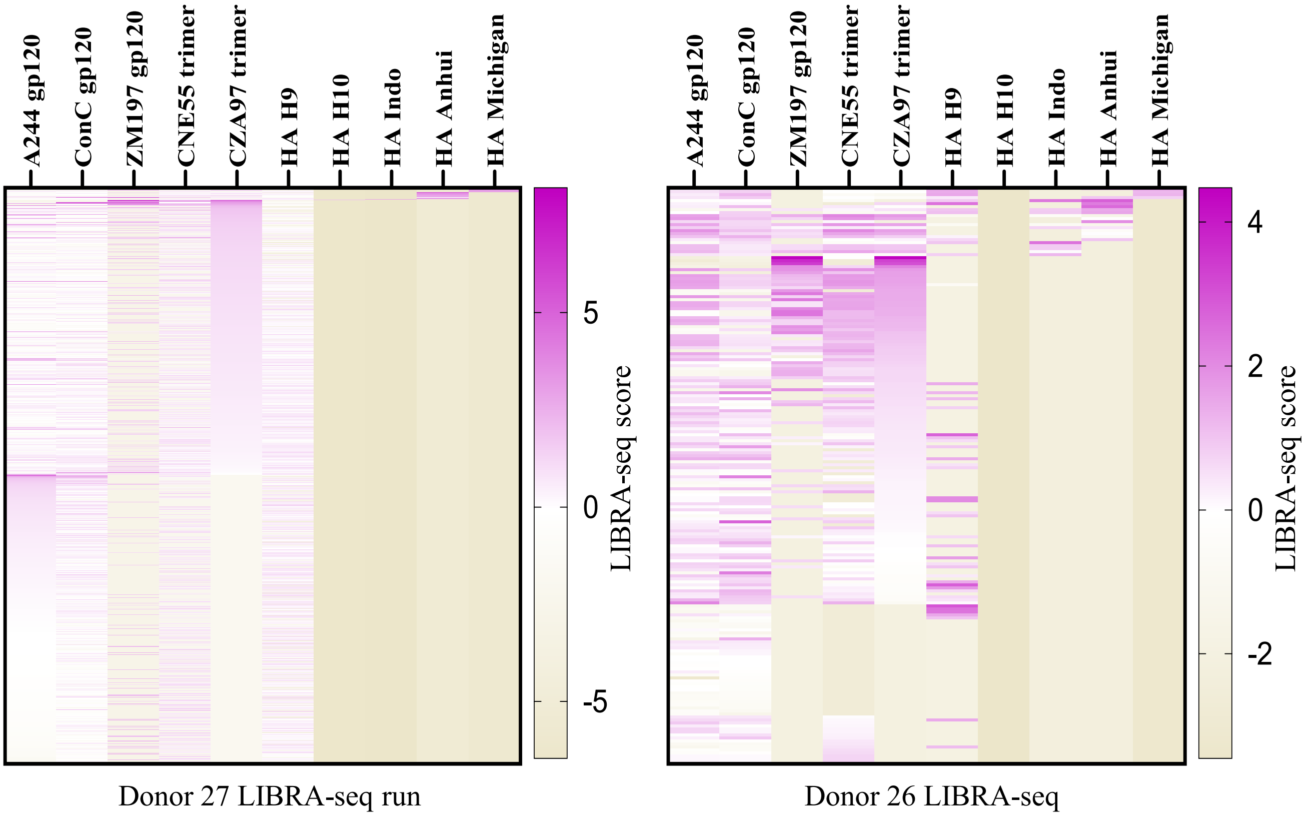

Supplementary Figure 3. Identification of HIV-1 Monomer and Trimer Specific B Cells using LIBRA-seq. LIBRA-seq score information for all cells from N27 LIBRA-seq run and N26 LIBRA-seq run. LIBRA-seq score information for each antigen is displayed on a scale from light yellow(low)-white-purple(high).

(A)

mAb	VH Gene	VH % Identity	CDRH3 length	VDJ Junction	VL Gene	VL % Identity	CDRL3 length	VJ Junction
4487-1	IGHV3-33	94.79	17	CARDPAAGRSYYYYYVDVW	IGLV1-40	96.88	11	CQSYDSNLSGVVF
4487-2	IGHV4-4	90.88	17	CARMFYESSTGYPSWFDPW	IGKV4-1	90.01	9	CQQYFYTPWTF
4487-3	IGHV2-5	90.03	16	CAYRLEHPSNWYDYFHHW	IGKV1-5	90.68	9	CQQYNFHSPTF
4487-4	IGHV1-46	75.35	15	CATSSGVVSLRSAFDIW	IGLV2-23	85.07	10	CCSYVGSNTWVF
4487-5	IGHV1-69	82.29	16	CARDGLRLNFYYYGMDVW	IGKV3-20	86.88	9	CQQNGESPRMF
4513-11	IGHV1-69	86.11	20	CARVGQPKFFERSSDGEHFQHW	IGKV1-5	85.3	9	CHQYSRYPWTF
4513-12	IGHV4-39	92.44	18	CARDVRMRSGRPRQYCMDVW	IGKV3-20	95.39	10	CQQYDTSPAVTF
4513-14	IGHV1-69	79.17	20	CAKDWSFDDDGGAYVPSAMDVW	IGKV3-20	89.72	9	CQQYGSSPFTF
4513-15	IGHV3-48	100	18	CARDFYYDSSGYYPDAFDIW	IGKV3-15	1	8	CQQYNNGRTF
4513-16	IGHV3-23	93.75	12	CARDIGLGRGHDYW	IGLV2-14	94.44	11	CSSYIAYNILEVF
4513-17	IGHV1-69	84.72	18	CARGRHSVVEPAAKYRFDTW	IGKV1-39	91.4	9	CQQSYSTPPTF

(B)

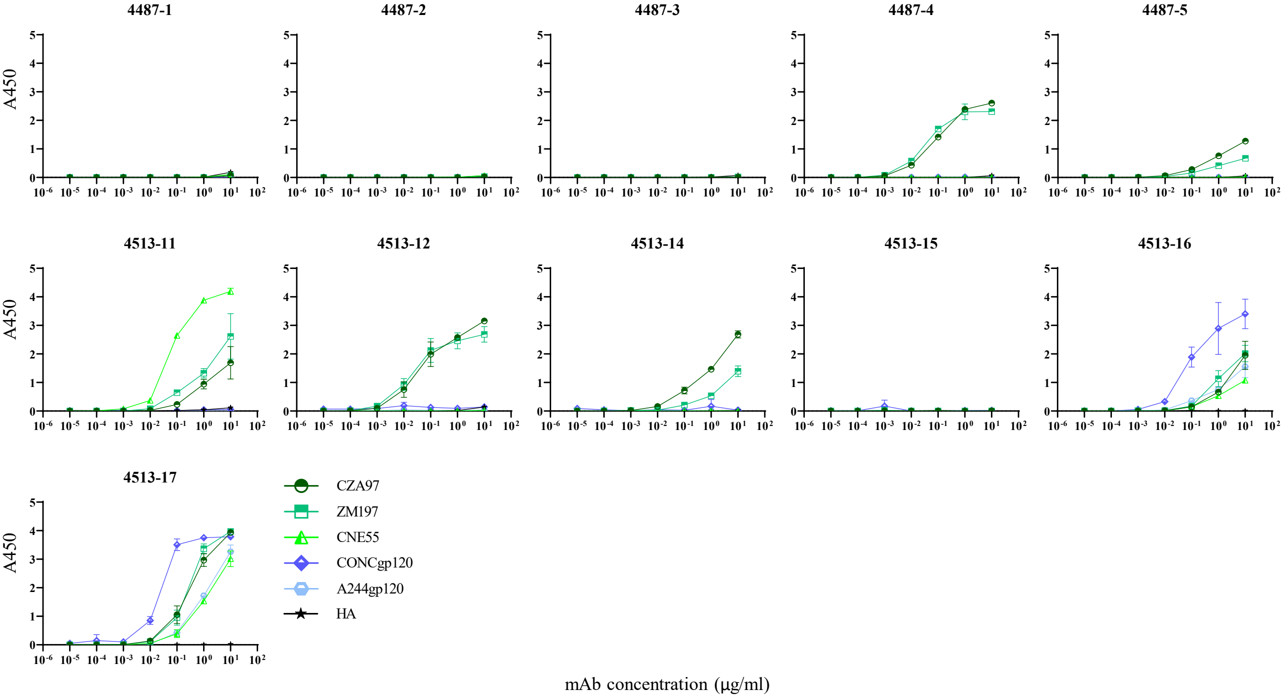

Supplementary Figure 4. Validation and characterization of monomer and trimer specific antibodies discovered through LIBRA-seq. (A) Sequence characteristics for candidate antibodies. Percent identity was calculated at the nucleotide level, and CDR length and sequences are displayed at the amino acid level. **(B)** ELISA binding curves for binding to HIV-1 trimeric and monomeric proteins are shown for candidate antibodies. Flu HA protein used as a negative control antigen. Absorbance values at 450nm shown on Y-axis. Antibody concentration in μg/ml shown on the X-axis.
